# Topography of Lipid Droplet-Associated Proteins: Insights from Freeze-Fracture Replica Immunogold Labeling

**DOI:** 10.1155/2011/409371

**Published:** 2010-12-26

**Authors:** Horst Robenek, Insa Buers, Mirko J. Robenek, Oliver Hofnagel, Anneke Ruebel, David Troyer, Nicholas J. Severs

**Affiliations:** ^1^Leibniz Institute for Arteriosclerosis Research, University Münster, Domagkstr. 3, 48419 Münster, Germany; ^2^Heart and Lung Institute, Imperial College, London SW3 6LY, UK

## Abstract

Lipid droplets are not merely storage depots for superfluous intracellular lipids in times of hyperlipidemic stress, but metabolically active organelles involved in cellular homeostasis. Our concepts on the metabolic functions of lipid droplets have come from studies on lipid droplet-associated proteins. This realization has made the study of proteins, such as PAT family proteins, caveolins, and several others that are targeted to lipid droplets, an intriguing and rapidly developing area of intensive inquiry. Our existing understanding of the structure, protein organization, and biogenesis of the lipid droplet has relied heavily on microscopical techniques that lack resolution and the ability to preserve native cellular and protein composition. Freeze-fracture replica immunogold labeling overcomes these disadvantages and can be used to define at high resolution the precise location of lipid droplet-associated proteins. In this paper illustrative examples of how freeze-fracture immunocytochemistry has contributed to our understanding of the spatial organization in the membrane plane and function of PAT family proteins and caveolin-1 are presented. By revisiting the lipid droplet with freeze-fracture immunocytochemistry, new perspectives have emerged which challenge prevailing concepts of lipid droplet biology and may hopefully provide a timely impulse for many ongoing studies.

## 1. Introduction

Freeze-fracture electron microscopy was established as a major technique in ultrastructure research over 30 years ago. It is a technique that revolutionized our understanding of membrane structure [1–3]. When biological specimens are in the frozen state, cellular bilayer lipid membranes have a plane of weakness in their hydrophobic interior; so if the sample is fractured, the fracture plane will split the membrane into half-membrane leaflets, each corresponding to a phospholipid monolayer with associated proteins. The result is a three-dimensional perspective of the membranous organization of the cell, with *en face* views of the membrane interior. These details are made visible in the electron microscope by making a very fine platinum-carbon replica of the fracture plane. The platinum is evaporated onto the specimen at an angle, so that it is deposited in varying thickness according to the topography at the fractured surface. In this way, high resolution details of membrane structure are revealed that cannot be seen by other techniques. By freeze-fracture, the distribution and organization of integral membrane proteins (seen as intramembrane particles), and other specialized features, are rendered visible in the membrane.

The freeze-fracture technique revolutionized the way we look at membranes, and its contribution to our knowledge of membrane structure was unrivalled, but one limitation remained—the need to identify the chemical nature of the structural components visualized. Without this knowledge, the functions of newly discovered structural features remained speculative. Thus, the combination of cytochemistry with freeze-fracture was a widely recognized goal. The technical challenges involved in developing effective techniques in freeze-fracture cytochemistry were considerable and took several decades to be overcome. 

Of all the techniques in freeze-fracture cytochemistry attempted and tested over the last three decades [4–7], the freeze-fracture replica immunolabeling (FRIL) technique has proven to be the most successful, and is currently having a substantial impact in solving questions in cell biology that have hitherto been impossible to address with other ultrastructural, cell biological, or molecular approaches [8–10].

 To illustrate the potential of this approach, we present here image data on the localization of caveolin-1 and PAT family proteins (the collective term for perilipin, adipophilin and TIP 47), which are associated with lipid droplets. By revisiting the lipid droplet and its associated proteins with FRIL, new perspectives have emerged which challenge prevailing concepts on a number of fronts and open up new questions for future research.

## 2. Results and Discussion

### 2.1. Standard Freeze-Fracture

**Figure 1:**
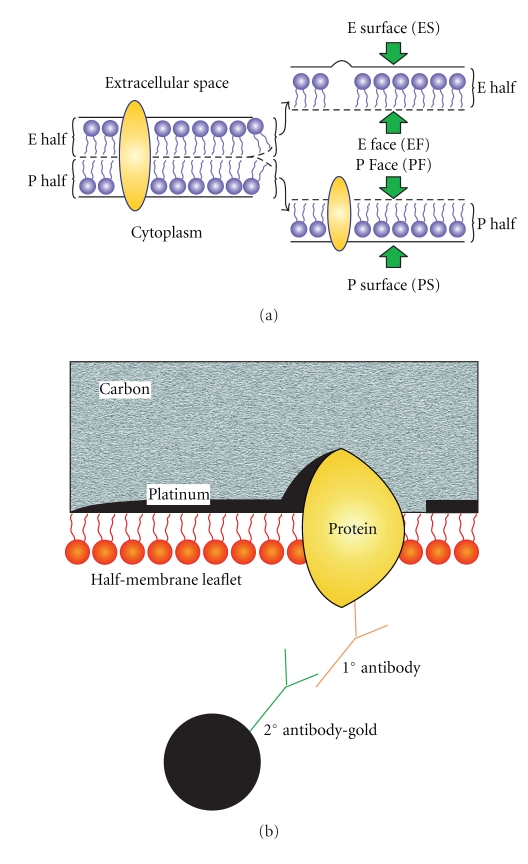
Nomenclature for describing the aspects of membranes revealed by freeze-fracture. (a) Cell samples are rapidly frozen and fractured. The freeze-fracture process splits the membrane exposing the fracture faces. The membrane comprises a lipid bilayer with intercalated proteins. The half-membrane leaflet adjacent to the extracellular space is termed the E half; that adjacent to the protoplasm is termed the P half. The term “fracture face” is reserved for the interior views of membranes exposed by freeze fracturing, while the term “surface” is used for the true, natural surfaces of the membrane. The fracture face of the P half is thus termed the P face (or PF), while that of the E half is termed the E face (or EF). The true surfaces of the membrane are correspondingly designated the P surface and the E surface (PS and ES), respectively. (b) Close-up view of the final product in FRIL as viewed in the transmission electron microscope. A platinum-carbon replica is made of the fractured specimen. The replica is treated with SDS to remove the cellular components apart from those attached directly to the replica. Proteins still attached to the replica are then immunogold-labeled. On examination in the electron microscope, the electron dense gold label is clearly visible against the replica, marking the target molecule in the plane of the membrane. The proteins embedded in the replica are detected using a primary antibody followed by a secondary antibody coupled to a colloidal gold marker.

An understanding of the principles and methodology of freeze-fracture cytochemistry requires a basic knowledge of how standard freeze-fracture works. The utility of freeze-fracture depends critically on the tendency of the fracture plane to follow a plane of weakness in the hydrophobic interior of frozen membranes, splitting them into half-membrane leaflets (Figure 1(a)). There are four essential steps in making a standard freeze-fracture replica: (1) rapid freezing of the specimen; in routine application, the freezing step is often preceded by pretreatment with glutaraldehyde fixation and glycerol cryoprotection (2) fracturing it at low temperature (−100°C or lower) (3) making a replica of the newly exposed frozen surface by vacuum-deposition of platinum and carbon (4) cleaning the replica to remove the biological material. For a detailed protocol on how to carry out freeze-fracture, see Severs [2]. The replica is then examined in the transmission electron microscope. At high magnification, details of membrane structure are seen at macromolecular resolution. In particular, the distribution and organization of integral membrane proteins (seen as intramembrane particles) are viewed in the membrane plane.

### 2.2. Freeze-Fracture Replica Immunolabeling

An important landmark in the progress to develop an effective technique in freeze-fracture replica immunolabeling was the introduction of colloidal gold labeling to electron microscopy in the early 1980s. Gold particles, because of their high electron density and small size, were quickly recognized to be ideal markers to use in conjunction with replicas. For the purposes of the present paper, a key foundation to the development of FRIL was the recognition that half-membrane leaflets could be retained attached to the platinum-carbon replica without significantly interfering with visibility of structure, and that these could provide a source of epitopes for immunogold labeling [8, 9, 11]. 

 The principle of the FRIL technique is explained in Figure 1(b). The specimen is rapidly frozen, freeze-fractured and platinum-carbon replicas made following the standard protocol used for freeze-fracture electron microscopy. However, instead of removing the biological material from the replica with bleach or acids as in the conventional technique, the replica is treated with sodium dodecyl sulphate (SDS) [8, 9]. The SDS removes the bulk of the biological material from the replica so that structure is visible by electron microscopy, leaving a single lipid monolayer and associated integral and surface proteins adherent to the replica. This remaining layer is so thin that it does not obstruct the electron beam. The proteins are then localized by immunogold labeling. The spatial distribution of the targeted epitopes can be viewed superimposed upon the membrane interior. Both membrane leaflets of the plasma membrane and those of intracellular membranes are accessible to labeling.

### 2.3. Freeze-Fracture Nomenclature

Understanding the topological relationships between the different membrane systems of the cell and the conventions used in describing freeze-fractured membranes is essential for interpreting freeze-fractured specimens. To apply the FRIL technique and interpret results, the practitioner has to be acquainted with the nomenclature used for describing standard freeze-fracture images [12]. This nomenclature is best explained by first thinking of the membrane as consisting of two halves—a P half which lies adjacent to the protoplasm (i.e., cytoplasm or nucleoplasm), and an E half which lies adjacent to the extracellular, exoplasmic or endoplasmic space. The term fracture face is applied to the interior views exposed as a result of splitting the membrane by freeze-fracture. The fracture face of the P half is thus termed the P face, while that of the E half is termed E face. The term surface is reserved for the true surface of the membrane. The true surfaces of the P half and the E half of the membranes are termed the P surface and the E surface, respectively (Figure 1). 

 To apply this nomenclature to the lipid droplet requires some adaptation because of the unusual structure of this organelle. Standard freeze-fracture nomenclature cannot be applied to lipid droplets as these organelles do not have a limiting phospholipid bilayer membrane as normally found enveloping organelles. The lipid droplet consists of a hydrophobic neutral lipid core surrounded by a single monolayer of phospholipids. 

**Figure 2:**
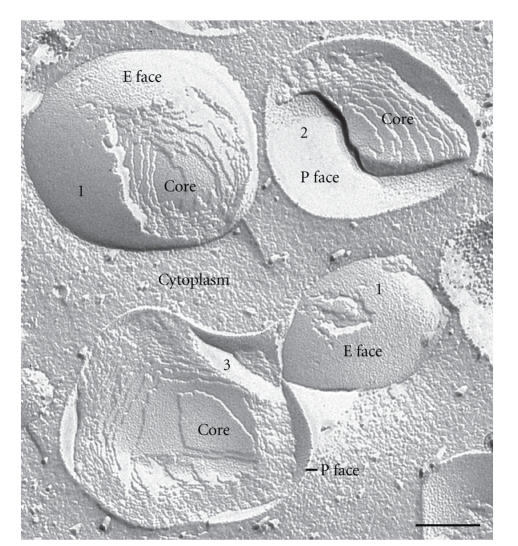
Freeze-fracture of lipid droplets. In freeze-fracture, lipid droplets have a unique smooth appearance enabling their unambiguous discrimination from other organelles. Three different types of view of the droplet are seen with this technique. (1) The fracture may travel upwards and over the droplet to give a convex fracture, (2) downwards and under to give a concave fracture, (3) or the droplet may be cross-fractured to give what is essentially a cross-section of the core. In concave fracture, the enveloping outer phospholipid monolayer is seen en face (P face); convex fractures give mirror image (complementary, E face) views. In practice, the three alternative fracture paths often occur in combination; concavely fractured droplets often include a portion of the core from small regions of cross-fracture, and some fractures skip along successive layers of the lipid revealing a multilayered onion-like appearance. Bar: 0.2 *μ*m.

 Concavely fractured droplets thus show an aspect of this monolayer that is equivalent to the P face of a normal membrane. The complementary aspect, revealed in convex fractures, would be termed an E face in the case of a normal lipid bilayer, but this designation is not appropriate since there is no E half to the enveloping structure, hence convex fractures show the outer-facing aspect of the lipid core. Some fractures through lipid droplets expose multilayers of lipid, giving an onion-like morphology. Lipid droplets that are cross-fractured reveal a simple homogeneous content or stacked internal fracture faces (Figure 2).

### 2.4. Compartmentalization of Lipid Droplet-Associated Proteins

Lipid droplets are versatile metabolically active organelles of intracellular lipid metabolism [13–15]. They consist of a core of hydrophobic neutral lipids (triglycerides and sterol esters) surrounded by an envelope of polar lipids (phospholipids, cholesterol, and fatty acids). The lipid droplet envelope is thought to be essentially a membrane monolayer derived from the cytoplasmic leaflet of the ER membrane [16], but hard-and-fast evidence for this origin is sorely lacking. Associated with lipid droplets are characteristic proteins including the PAT-family proteins (perilipin, adipophilin, and TIP 47, and others) and the caveolins, and recent proteomic analyses have revealed a myriad of as-yet-to-be confirmed or identified protein species including histones in lipid droplets [17]. Specific lipid droplet proteins regulate various lipid droplet functions, and abnormalilties in the complement of lipid droplet proteins are hence directly reflected in diseases like obesity, diabetes, atherosclerosis, and disorders with lipid storage dysfunction [18].

Where are all the lipid droplet proteins then? For one thing, they are inserted into the droplet envelope. This location has been verified many times by fluorescence and electron microscopy. It is the expected and accepted location, because lipid droplet proteins and envelope lipids are both amphipathic and can conveniently mingle in the envelope. And after all, we are used to proteins integrated into cell membranes—although, as explained above, the droplet envelope is not a membrane at all, only half of one. Until recently, PAT family proteins and caveolins were considered to be associated exclusively with the droplet envelope, not with the core or other organelles. Consequently, regulation of intracellular lipid metabolism was hypothesized to occur at the interface between the lipid droplet and the cytosol [19]. As we will see below, however, the application of FRIL has overturned these notions.

Perilipin associates with lipid droplets in adipocytes and steroidogenic cells. Adipophilin is an ubiquitously expressed lipid droplet-associated protein of all mammalian cell types [20–22]. TIP 47 was originally described as a cytosolic protein which binds to the cytoplasmic domains of the cation-dependent and cation-independent mannose 6-phosphate receptor. Subsequent studies reported TIP 47 at the lipid droplet surface of HeLa cells and other cell types. Our recent studies revealed that TIP 47 is not restricted to cytosolic compartments but is also localized in the lipid droplet core and in the plasma membrane of macrophages in a similar manner to that found with adipophilin and perilipin. The function of adipophilin and TIP 47 in foam cell formation is only partly understood [23]. A number of other proteins have been reported in association with lipid droplets, in particular caveolin-1, a protein best known as a structural component of caveolae [24]. 

**Figure 3:**
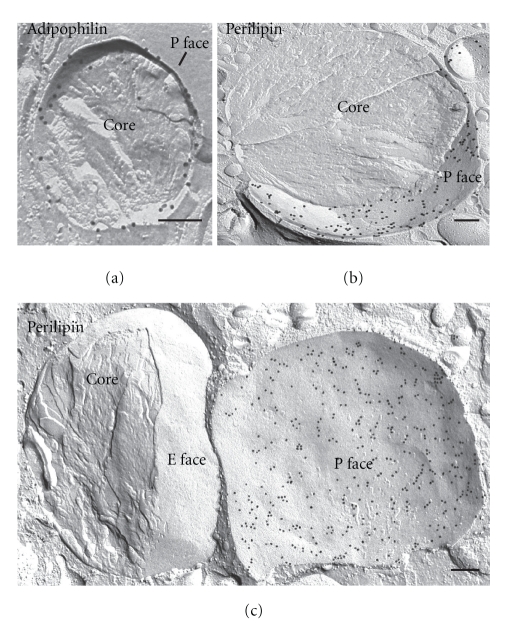
FRIL demonstrates the presence of adipophilin and perilipin in lipid droplets. Examples in which adipophilin (a) and perilipin (b) label is seen in the outer phospholipid monolayers (P face) of the lipid droplet. The example in (c) illustrates a convexly and a concavely fractured lipid droplet. Perilipin is localized in the P face of the lipid droplet monolayer, whereas the E face is completely devoid of label. ((a) from a lipid-laden macrophage; (b) and (c) from lipid-laden adipocytes). Bars: 0.2 *μ*m.

**Figure 4:**
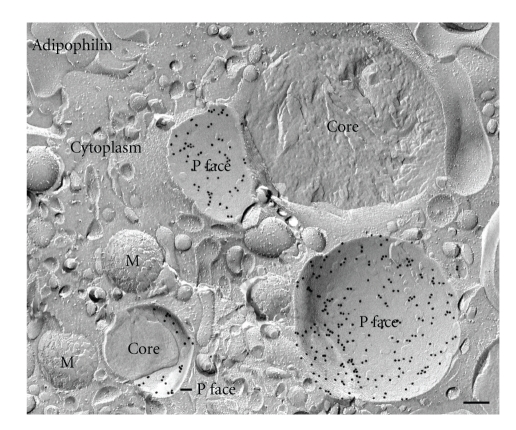
Survey freeze-fracture view of a lipid-laden macrophage immunogold-labeled for adipophilin. Prominent label is seen in the outer phospholipid monolayer (P face) of the lipid droplets. M: mitochondria. Bar: 0.2 *μ*m.

The advantages of FRIL are illustrated in Figures 3 and 4. Electron microscopy of immunogold-labeled replicas localize adipophilin (Figures 3(a) and (4) and perilipin (Figures 3(b) and 3(c))) to the periphery of the lipid droplet. While cross-fractured lipid droplets reveal a peripheral ring of label (Figure 3(a)), in concavely fractured droplets *en face* views of this surface label are seen, allowing its distribution and extent to be appreciated (Figures 3(b) and 3(c)). The convexly fractured lipid droplets are devoid of label (Figure 3(c)).  Figure 4 illustrates a survey freeze-fracture view of lipid droplets in a lipid-laden macrophage after immunogold labeling for adipophilin. Results such as these, in which the PAT family proteins are localized to the outer surface of the lipid droplet, suitably position these proteins to function in the mobilization or accretion of lipids, at the interface between the droplet and the cytoplasm [17].

**Figure 5:**
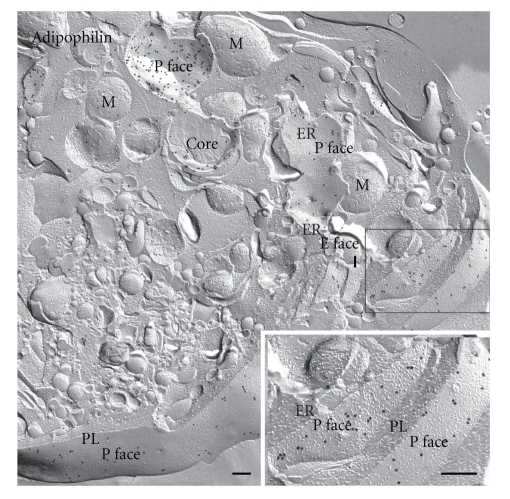
Adipophilin in cellular membranes. Survey freeze-fracture view of a lipid-laden macrophage immunogold-labeled for adipophilin. Apart from positive labeling in the periphery of lipid droplets prominent label is seen in the P face of the plasma membrane (PL) and ER membranes. The E faces of the ER, mitochondrial and vesicle membranes are devoid of label. Inset: higher magnification of the P face of ER and plasma membrane. M mitochondria. Bar: 0.2 *μ*m.

**Figure 6:**
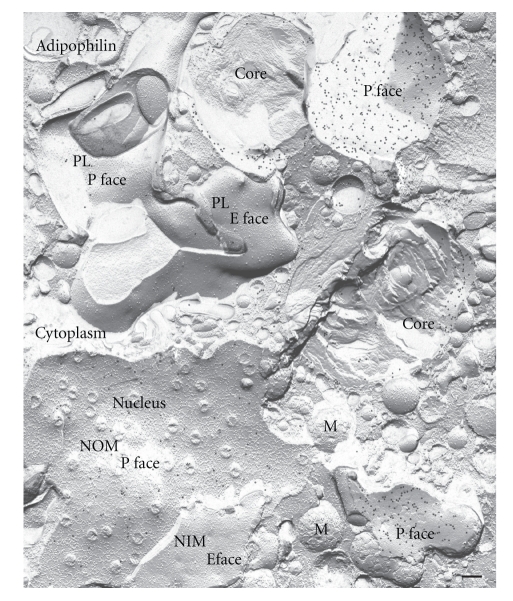
Freeze-fractured nuclear membrane in a lipid-laden macrophage after immunogold labeling for adipophilin. Apart from positive labeling of lipid droplets, the P face of the outer nuclear membrane (NOM) is prominently labeled. The E face of the outer nuclear membrane and both fracture faces of the inner nuclear membrane (NIM) are typically devoid of label. Bar: 0.2 *μ*m.

**Figure 7:**
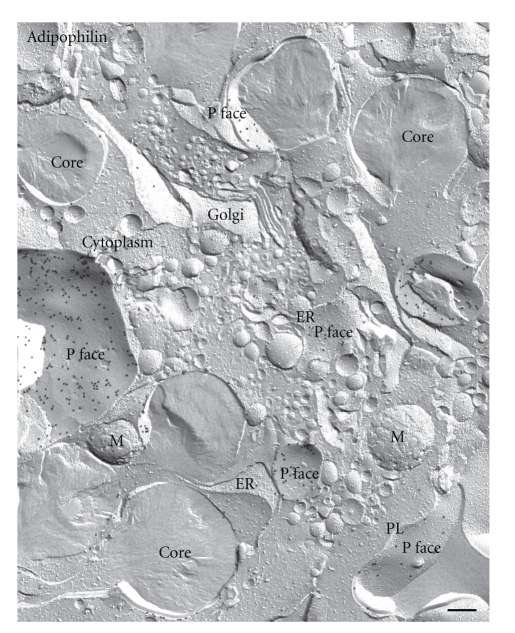
Freeze-fracture overview of organelles in a lipid-laden macrophage after labeling for adipophilin. Gold particles marking the presence of adipophilin can be seen in abundance in the outer phospholipid monolayer (P face) of lipid droplets, ER membrane and plasma membrane (PL). The Golgi apparatus (Golgi) is devoid of label. M mitochondria. Bar: 0.2 *μ*m.

However, apart from positive labeling in the periphery of lipid droplets prominent label for adipophilin in lipid-laden macrophages is also apparent in ER membranes and plasma membranes (Figure 5). The labeling in these membranes is confined to the P face; no label is detected in the E face. Adipophilin label is also seen in the P-face of the outer nuclear membrane (Figure 6) with which the ER membrane P face is continuous. No label is apparent in the E face of the outer nuclear membrane or in either fracture face of the inner nuclear membrane. The membranes of the Golgi apparatus, mitochondria, and vesicles were consistently devoid of label (Figure 7).

**Figure 8:**
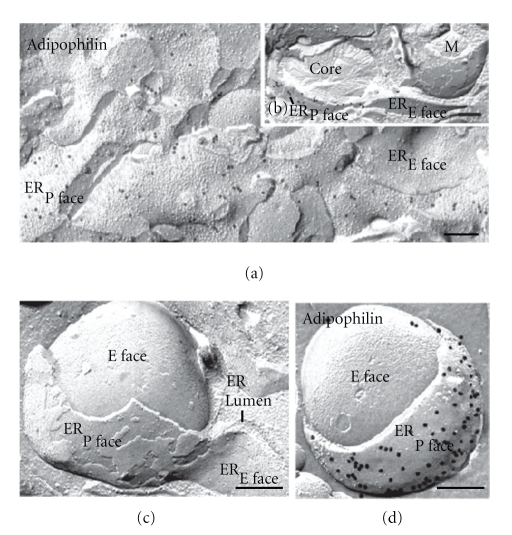
Freeze-fracture views of lipid droplet and ER membrane associations in lipid-laden macrophages immunogold-labeled for adipophilin. (a) ER membranes are visible in both P face and E face views. Moderate labeling is seen in the P faces of the ER membranes. (b) Lipid droplet closely associated with ER membranes. Abundant gold label marks the presence of adipophilin in the ER membrane immediately adjacent to the lipid droplet. In contrast, ER membranes adjacent to mitochondria (M) are devoid of label. (c) Lipid droplet situated in a cup formed from ER membranes. The lipid droplet has been convexly fractured and lies beneath (i.e., adjacent to and not within) both ER membranes exposed. (d) Similar view to (c) but with labeling for adipophilin using the FRIL technique. Abundant gold label marks the presence of adipophilin in the ER membrane (P face) immediately adjacent to the lipid droplet. Bars: 0.2 *μ*m.

Apart from disclosing differential labeling in the leaflets of the various membrane systems of the cell, freeze-fracture reveals three-dimensional aspects of intracellular membranes, permitting unique views of the spatial relationship of these membranes. Such perspectives enable the nature of the intimate association between lipid droplets and ER segments to be appreciated (Figure 8). Figure 8(a) presents freeze-fracture images of the sites of ER-lipid-droplet association. Freeze-fracture immunocytochemistry reveals distinctive labeling patterns for adipophilin at the sites of lipid droplet association. Abundant labeling for adipophilin is seen in the P face of the ER membrane that lies closest to the lipid droplet; no label is detected in the E face of the partner ER membrane distant from the droplet. 

 The mechanism of lipid droplet formation that has gained general acceptance holds that neutral lipids accumulate within the lipid bilayer of the ER membrane from where they are budded-off, enclosed by a protein-bearing phospholipid monolayer originating from the cytoplasmic monolayer of the ER membrane, to give a cytoplasmic lipid droplet [16]. How the lipids are channeled to budding domains, how budding occurs mechanistically, and how the directionality of the budding is achieved are not understood. Indeed, little direct experimental evidence supports the budding model, and alternative models have been proposed [20, 25]. An alternative model was recently put forth in which lipid droplets are excised from both leaflets of the ER membrane bilayer as a bicelle [26]. Another model proposed by Walther and Farese [20] is called vesicular budding. Droplets are initially formed within small bilayer vesicles, utilizing the machinery of vesicle formation of the secretory pathway. This might occur in a specialized domain of ER that is distinct from vesicular transport and dedicated to lipid synthesis. Newly formed lipids might fill the bilayer of existing membrane vesicles, effectively filling the vesicles with neutral lipids. In a variation of this model the ER bilayer is filled with neutral lipids and the resulting bulge does not detach from the ER.

Freeze-fracture, by permitting unique three-dimensional views of the spatial relationship of membranes and organelles, demonstrates unequivocally that at sites of close association, the lipid droplet is not situated within the ER membrane, but adjacent to it. Both ER membranes clearly lie external to and follow the contour of the lipid droplet, enclosing it in a manner akin to an egg-cup (the ER) holding an egg (the lipid droplet) (Figure 8(c)). FRIL further demonstrates that adipophilin is concentrated in prominent clusters in the P half of the ER membrane at the site of the closely opposed lipid droplet (Figures 8(b) and 8(d)), as well as in the lipid droplet surface opposed to the ER. Adipophilin is thus strategically placed to play a role in lipid droplet growth by facilitating lipid transfer from the ER to the droplet. The evidence from these studies indicates that lipid droplets originate and develop adjacent and external to specialized domains of the ER membrane enriched in adipophilin, not within the bilayer of the ER as previously supposed.

**Figure 9:**
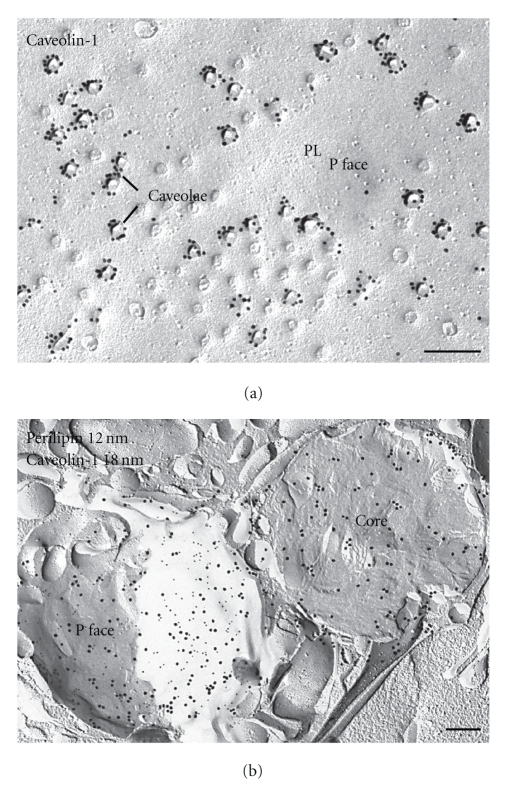
Distribution of caveolin-1 and perilipin in adipocytes. (a) Caveolae appear as dimples in the P face of the plasma membrane of adipocytes. Gold particles label cavolin-1 in the P face at caveolae. Caveolin-1 labeling is found mainly at the rims of deep caveolae. Apart from positive labeling of caveolae in the plasma membrane, abundant label of caveolin-1 is found in the lipid droplet. (b) Example of immunogold labeling of perilipin (12 nm gold) and caveolin-1 (18 nm gold) in two lipid droplets of the same cell. One droplet shows colocalization of both proteins in the outer monolayer (P face) in almost equal amounts, whereas the other is cross-fractured and the core contains almost exclusively caveolin-1 label. Bars: 0.2 *μ*m.

 The caveolins, originally described as ubiquitous integral proteins of cell surface pits called caveolae, are of immense physiologic interest, because they are involved in signal transduction and cellular cholesterol homeostasis [27]. Just how caveolins assist in these capacities is currently an area of particularly active research. The best studied caveolin, caveolin-1, cycles constitutively between the plasma membrane and several intracellular compartments [28]. Cavelion-1 binds directly to cholesterol and is believed to participate facultatively in the bidirectional shuttling of free cholesterol between the cell surface and the various intracellular compartments, including ER, Golgi, and particularly lipid droplets. At its main location at the cell surface, caveolin-1 is an unusual protein, because both of its hydrophilic ends project into the cytosol, while the intervening region is anchored into the lipid phase of the membrane bilayer. Caveolin-1 does not entirely span the lipid bilayer, but is confined essentially to one leaflet of the membrane. After immunolabeling replicas with anticaveolin-1, caveolin-1 label is found in the P face of the plasma membrane of adipocytes (Figure 9(a)). The E face is completely unlabeled. Deep caveolae are generally strongly marked on their rims, whereas labeling of shallow caveolae and morphologically undifferentiated regions of the plasma membrane is usually weaker.

Immunolabeling shows that caveolin-1 is also present in intracellular organelles of adipocytes including lipid droplets. The prevailing wisdom holds that caveolin-1 and also the PAT family proteins are confined exclusively to the droplet surface [29–31]. FRIL, however, demonstrates that these proteins are distributed not only in the surface but also throughout the lipid droplet core. Double labeling of perilipin and caveolin-1 reveals extreme heterogeneity with respect to the distribution of these two lipid droplet-associated proteins in lipid droplets of adipocytes.

Figure 9(b) illustrates an example of one lipid droplet in which the fracture has exposed the outermost monolayer (P face) and another that has been cross-fractured in the same cell. The P face exhibits colocalization of perilipin and caveolin-1, but the core of the other droplet contains almost exclusively caveolin-1.

The key finding of these studies is that in mature lipid droplets, lipid droplet-associated proteins clearly gain access to the successive interior lamellae and the amorphous interior of the lipid droplets, as indicated by the presence of specific label in these zones. Labeling of the droplet-associated proteins in lipid droplets is far from being confined to the P face. Other proteins have also been found deep inside the lipid-droplet core. Several independent methods have been used to detect them there. Proteins now acknowledged to be inside the lipid droplet core are PAT family proteins [32, 33], caveolins [34], flotillins [35] and cyclooxygenase [36], among others [37, 38].

Reports of lipid droplet proteins in the lipid droplet core appear to be studiously neglected. Why? Because the presence of lipid droplet proteins in the core raises several disconcerting questions. First, how do the proteins get into the core in the first place? This probably hinges on how lipid droplets form at the ER. Lipid droplet proteins could gain access to the core if both neutral lipids and lipid droplet proteins in the ER are transferred across the phospholipid layer of the droplet envelope during lipid droplet biogenesis. Indeed, one compelling hypothesis suggests that lipid transfer between the ER and the droplet does take place across the droplet envelope and depends on the recruitment of Rab 18 to the droplet in response to stimulation of lipolysis by *β*-adrenergic agonists. Furthermore, at least one PAT protein, adipophilin, which is present in ER, seems to be conferred to the nascent droplet from adjacent clusters of adipophilin in the cytoplasmic leaflet of the ER membrane. Second, how can those amphipathic proteins ever be accommodated among the hydrophobic lipid molecules of the core? Freeze-fracture studies reveal that the lipids of the cores are preferentially arranged in thin layers like the leaves of an onion. Therefore, one explanation is that lipid droplet proteins may be forced into place onto the interfaces between the internal lamellar structures of the core during droplet biogenesis, even though they do not normally mix with neutral lipids. Taken together, these two explanations would mean that regulation of intracellular lipid metabolism occurs between the lipid droplet and the ER rather than between droplet and cytosol as currently preached. 

How much longer are we going to ignore the presence of lipid droplet proteins in lipid droplet cores? After all, studying how they get there might help us to appreciate better how lipids and proteins are exchanged across phospholipid barriers, how the ER takes part in lipid droplet biogenesis, and how lipid droplet-associated proteins regulate intracellular lipid metabolism in health and disease.

**Figure 10:**
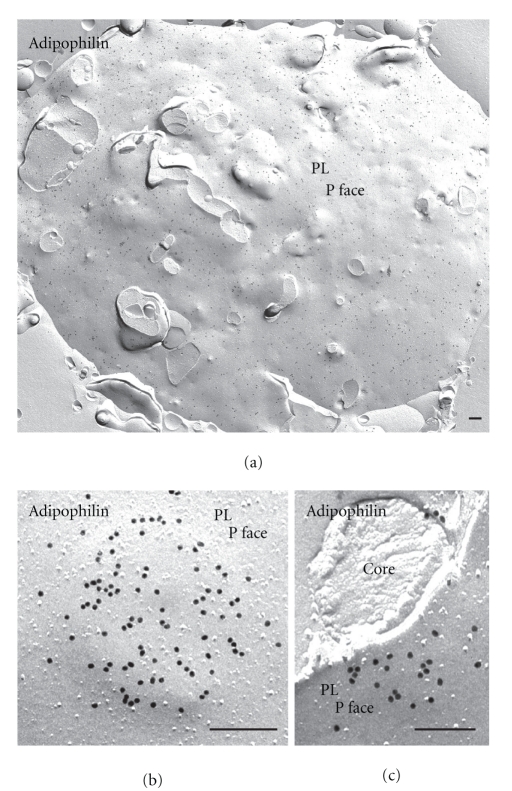
FRIL images demonstrating that PAT family proteins are present in the plasma membrane. (a) View of the plasma membrane (PL, P face) of a normal cultured macrophage after labeling for adipophilin. The adipophilin is widely distributed throughout the membrane. (b) Upon lipid loading, the adipophilin becomes clustered in elevated domains in the plasma membrane. (c) Fractures that penetrate beneath the plasma membrane demonstrate that lipid droplets lie beneath the elevated adipophilin-rich domains. Bars: 0.2 *μ*m.

We have seen that PAT family proteins are not, as previously supposed, specific to lipid droplets but also occur in specialized ER membranes, adjacent to the droplet at the proposed sites of biogenesis and growth. Through application of FRIL, however, it is clear that sites similar to those identified in the ER are also present in other cellular locations. Another area to which FRIL has shed new light is the finding of PAT family proteins as integral components of the plasma membrane of macrophages and adipocytes. Under normal culture conditions, these proteins are dispersed in the P half of the plasma membrane (Figure 10(a)). Stimulation of lipid droplet formation by incubation of the cells with acetylated low-density lipoprotein leads to clustering of the PAT family proteins in raised plasma membrane domains (Figure 10(b)). Fractures penetrating beneath the plasma membrane demonstrate that lipid droplets are closely opposed to these domains (Figure 10(c)).

The discovery by FRIL of PAT family proteins in the plasma membrane came as a surprise, given that no evidence for such a localization was apparent from immunofluoresence microscopy or subcellular fractionation. However, in normal cells, because FRIL shows perilipin and adipophilin to be widely dispersed in the plasma membrane, the proteins are unlikely to be above the concentration threshold required for clear detection by immunofluorescence. Once clustered, the potential for detectability is increased but as the clusters are extremely closely opposed to lipid droplets, immunofluorescence microscopy has insufficient resolution to determine whether any labeling observed derives from the lipid droplet itself or the immediately adjacent plasma membrane domain. As perilipin and adipophilin are envisaged as characteristic proteins of the lipid droplet, it is understandable that any such signal observed would be assumed to derive from the lipid droplet. Similarly, lack of plasma membrane localization of adipophilin and perilipin in subcellular fractionation may arise in part from adherence of the closely opposed plasma membrane domains to lipid droplets during their isolation. 

 The relationship between lipid droplets and PAT family protein-enriched plasma membrane domains is strikingly similar to that of the lipid droplets and the ER membranes discussed above. At times of extreme lipid loading, the number of sites in the ER may be insufficient to cope with demand for lipid droplet synthesis and growth, and thus equivalent machinery in the plasma membrane may be brought into play. It may be speculated that aggregation of the PAT family proteins into plasma membrane assemblies may facilitate carrier-mediated lipid or fatty acid influx directly from the extracellular environment into the growing lipid droplet. Whatever the functional role of these aggregates is, the findings suggest a common cellular mechanism of intracellular lipid loading in the macrophage as part of the pathogenesis of atherosclerosis and in the adipocyte during development of obesity.

## 3. Conclusion

By revisiting the lipid droplet with freeze-fracture electron microscopy and the FRIL technique, new findings have emerged which challenge previous assumptions on where its associated proteins are localized within the cell and how these proteins target to lipid droplets, and on how lipid droplets form and grow. Our aim here has been to present an integrated survey of a range of these new findings with a view to stimulating debate on their functional significance. We are, of course, mindful that morphology alone does not explain functional processes. Equally, however, a knowledge of structure underpins and provides the framework for understanding function, and without that knowledge, functional assumptions may be led seriously astray. The unique advantages of FRIL have yielded new information on the structure, composition and protein organization of the lipid droplet. 

We know now that PAT family proteins and caveolins are not confined to the surface of the lipid droplet as previously believed, but pervade the droplet core. There is no irrefutable evidence for the widely held view that the lipid droplet is formed within the ER membrane bilayer; our finding that lipid droplets appear to develop enclosed by but external to specialized sites of the ER membrane bilayer that are enriched in adipophilin challenges the long held concept that they are formed within the ER membrane bilayer.

PAT family proteins are not specific to the lipid droplet, but are widely present in the plasma membrane where, under conditions of lipid loading, they adopt a similar configuration to the specialized sites in the ER. 

 The examples discussed illustrate the recent impact of the FRIL technique in advancing our understanding of selected aspects of lipid droplet biology. The information that this approach provides is unique and further exploitation of the FRIL technique may be expected as its scope and power become more widely appreciated.
